# Stereotactic Body Radiotherapy vs. Radiofrequency Ablation in the Treatment of Hepatocellular Carcinoma: A Meta-Analysis

**DOI:** 10.3389/fonc.2020.01639

**Published:** 2020-10-29

**Authors:** Yang-Xun Pan, Yi-Zhen Fu, Dan-Dan Hu, Qian Long, Jun-Cheng Wang, Mian Xi, Shi-Liang Liu, Li Xu, Meng-Zhong Liu, Min-Shan Chen, Yao-Jun Zhang

**Affiliations:** ^1^Sun Yat-sen University Cancer Center, State Key Laboratory of Oncology in South China, Collaborative Innovation Center for Cancer Medicine, Guangzhou, China; ^2^Department of Liver Surgery, Sun Yat-sen University Cancer Center, Guangzhou, China; ^3^Department of Oncology-Pathology, Karolinska Institutet, Stockholm, Sweden; ^4^Department of Experimental Research, Sun Yat-sen University Cancer Center, Guangzhou, China; ^5^Department of Radiation Oncology, Sun Yat-sen University Cancer Center, Guangzhou, China

**Keywords:** minimally invasive treatment, meta-analysis, hepatocellular carcinoma, stereotactic body radiotherapy, radiofrequency ablation

## Abstract

**Background:** Both stereotactic body radiotherapy (SBRT) and radiofrequency ablation (RFA) are effective local treatments for hepatocellular carcinoma (HCC), but whether RFA is superior to SBRT is still controversial. Therefore, we performed a meta-analysis to compare the treatment outcomes of SBRT with RFA as curable or bridge intention.

**Methods:** We searched online databases for studies that compared treatment outcomes for SBRT and RFA. Eligibility criteria included evaluation of local control, overall survival (OS), transplant rate, and post-transplant pathological necrosis.

**Results:** As no randomized clinical trials met the criteria, 10 retrospective studies with a total of 2,732 patients were included. Two studies were in favor of SBRT in local control, two studies preferred RFA in OS, and others reported comparable outcomes for both. SBRT demonstrated significantly higher 1- and 3-year local control than RFA [odds ratio (OR) 0.42, 95% CI 0.24–0.74, *P* = 0.003; and OR 0.54, 95% CI 0.37–0.80, *P* = 0.002, respectively]. However, SBRT reported significantly shorter 1- and 2-year OS (OR 1.52, 95% CI 1.21–1.90, *P* = 0.0003; and OR 1.66, 95% CI 1.38–2.01, *P* < 0.00001, respectively). As bridge treatment, no significant difference was shown in transplant rate and post-transplant pathological necrosis rate (OR 0.57, 95% CI 0.32–1.03, *P* = 0.060; and OR 0.49, 95% CI 0.13–1.82, *P* = 0.290, respectively).

**Conclusions:** This study demonstrates SBRT is able to complete a better local control for HCC than RFA, though the OS is inferior to RFA because of tumor burden or liver profiles of the enrolled studies. Well-designed, randomized, multicenter trials will be required to further investigate the conclusion.

## Introduction

Liver transplantation is the most beneficial therapy for early hepatocellular carcinoma (HCC) patients, as it removes both the tumor and the cirrhotic liver (1). However, given organ availability limitation, a number of patients who may benefit from this treatment have to stay on the waiting list for a long time. Therefore, stereotactic body radiotherapy (SBRT) and radiofrequency ablation (RFA) are offered as potential alternative local control modalities for patients in the waiting list (2, 3).

RFA induces coagulative necrosis of tumor through thermal effect and is the first-line treatment for small HCC (≤3 cm), providing comparable long-term outcome with resection (4). However, RFA has several contraindications, including large tumor size and lesions adjacent to major vessels or close to the liver hilum. The above circumstances may result in incomplete ablation, which potentially leads to worse prognosis (5–9).

SBRT is an advanced technology that delivers ablative radiation doses to tumors in a few fractions while minimizing the dose to normal liver tissue. Early results with SBRT have shown considerable local control even for large tumors or HCC ineligible for surgery (10). Moreover, SBRT has been frequently used as an alternative to RFA for small HCC patients with tumors near critical anatomical structures or major vessels due to the heat sink effect that can occur with RFA (11).

Recent publications have reported either comparable outcomes between the two treatment modalities or favorable outcomes for one to the other (3, 10, 12–19). Most of the published studies retrospectively reviewed clinical data in one single center; the results of these observational studies could have been strongly affected by several biases, and hence, the efficacy of these two treatments regarding disease control, long-term survival, and treatment related complications is ununified. With the absence of randomized data, meta-analysis might be able to draw a relative objective and reliable conclusion by integrating data from different clinical centers. The aim of this meta-analysis is mainly to compare the benefits of SBRT and RFA in the local progression (LP) control and overall survival (OS) in the treatment for HCC and to further help in clinical decision making.

## Materials and Methods

### Study Selection

The inclusion criteria of this meta-analysis were as follows: (1) diagnosed primary liver cancer definitively and patients diagnosed with HCC based on pathological evidence from fine-needle aspiration (FNA) or in the absence of biopsy evidence, based on imaging techniques including contrast-enhanced ultrasonography (CEUS), computed tomography (CT), and magnetic resonance imaging (MRI) companied with alpha-fetoprotein elevation; (2) no evidence of invasion into the major portal/hepatic vein branches or extrahepatic metastasis based on radiologic imaging; (3) patients without previous treatment of transcatheter arterial chemoembolization (TACE), surgery, chemotherapy, or other antitumor treatment; (4) documented indications for SBRT and RFA clearly; (5) either randomized controlled trials (RCTs) or retrospective studies were candidates; (6) patients of two groups with comparable basic clinical characters; and (7) studies with outcome information regarding LP rates, OS rates, and/or transplant rates.

Studies with following characteristics were excluded: (1) studies that did not report original data, including abstracts, case reports, expert opinions, editorials, reviews, or letters; (2) either group in the studies or combined other therapies; and (3) studies based on the same cohort.

### Search Strategy

A systematic online databases search of PubMed Central, Embase, Cochrane Library, and Google Scholar was separately conducted by two reviewers to identify all relevant availability of studies until August 26, 2019.

This meta-analysis was performed consistent with the Preferred Reporting Items for Systematic Reviews and Meta-Analyses (PRISMA) statement checklist. The subject headings (MeSH) search included “radiofrequency ablation,” “stereotactic radiation therapy,” and “hepatocellular carcinoma”; and keywords search was used, including “radiofrequency ablation,” “stereotactic body radiation therapy,” and “hepatocellular carcinoma.” These terms were used in different combinations. Only studies on humans and English-language studies were included. A manual research was performed by browsing all references of all identified studies. This progressed research was repeated to ensure to include the whole relevant studies. The research was completed by two reviewers before the data analysis independently (Y-XP and MX). If a study was controversial, the corresponding author was asked to judge (Y-JZ).

### Data Collection

Data were extracted from the included studies, including number of patients in the SBRT and RFA groups; age; gender; primary tumor size; number of tumors; median dose of SBRT; Child–Pugh class; median follow-up time; 1-, 2-, and 3-year LP rates; 1-, 2-, 3-, and 5-year OS rates; post-transplant necrosis rates; and time to liver transplant.

### Definitions

LP was defined as the recurrence of lesion in the treatment area by imaging studies. And LP time was the period from the initial treatment to the discovery of LP or last follow-up. The OS was defined the period from the date of initial treatment of the HCC to the date of death related to any cause or last follow-up. Transplant rate was the proportion of patients who received liver transplantation after SBRT or RFA therapies. Post-transplant pathological necrosis was evaluated by post-transplant pathology.

### Statistical Analysis and Synthesis

All analyses were performed with the help of statistical software, named Review Manager, version 5.3 (Nordic Cochrane Center; Oxford, England). For data evaluation, patients were assigned into two groups: the SBRT-treated group and the RFA-treated group. The odds ratio (OR) and/or hazard ratios (HRs) accompanying 95% confidence interval (95% CI) were calculated for dichotomous and univariable analysis outcomes in terms of LP, OS, and prognostic factor on treatment allocation. Meanwhile, we assessed the heterogeneity among trials according to the chi-squared (χ^2^) test including the inconsistency factor (*I*^2^). The heterogeneity was defined as a *P* < 0.05 or an *I*^2^ >40% (20). Given the small number of included studies, though the heterogeneity was not high, the random effects model was applied throughout to enhance the reliability of results. A potential publication bias was assessed by visually inspecting the Begg funnel plots in which the standard error (SE) of log OR or log HR was plotted against the OR or HR, respectively.

## Results

### Search Results and Quality Assessment

A total of 440 studies were identified for the first time from PubMed by the search strategy previously established, and 269 studies were identified *via* other sources or review. Subsequently, 11 studies were deleted for duplication with the help of Mendeley (Elsevier Inc., Atlanta, GA, USA). The titles and abstracts of 270 studies were then screened for inclusion. The full texts of 36 studies were read; and, finally, we included 10 non-RCT studies that met the present meta-analysis criteria (3, 10, 13–16, 18, 19, 21, 22). The details of the PRISMA flow diagram of the literature for meta-analysis is shown in Supplementary Figure 1 (23).

In the present analysis, three studies were based on already existing database (13, 15, 19), and the remaining seven studies were based on retrospective studies (3, 10, 16–18, 21, 22). Two studies conducted SBRT or RFA for transplant intent. Five studies were performed in the USA (10, 13, 15, 18, 19), two in Japan (12, 16), one in Canada (3), one in South Korea (17), and one in China (22). Out of 2,732 patients from the 10 included studies, 859 patients were classified into the SBRT group, and the rest of the 1,873 patients were classified into the RFA group. The Newcastle Ottawa Scale (NOS) (24) were used to assess the quality of non-randomized studies. Although the qualities of selections and outcomes were relatively appropriate in terms of each study, over 50% of included studies were medium-score studies because of inconsistent comparability. Therefore, we believed that the present meta-analysis possesses a medium class of quality (Supplementary Table 1).

Among included studies, two studies were in favor of SBRT on local control (10, 21), two studies preferred RFA on OS (15, 19), and others reported comparable outcomes between groups (3, 13, 16–18, 22). Notably, according to baseline characteristics, several studies enrolled patients in SBRT group were prone to suffer from larger tumor diameter (3, 10, 16, 19) and higher proportion of Child–Pugh C patients [(18); Table 1]. These groups were evaluated for therapeutic efficacy in treating HCC patients. The details of the studies included in the present meta-analysis are listed in Table 1.

**Table 1:** Characteristics of include studies.

**References**	**Design**	**Group**	**Number of patients**	**Number of tumor (1/≥2)**	**Age (years)**	**Sex (M/F)**	**Tumor size (cm)**	**Median dose (Gy)**	**Child-Pugh class (A/B/C)**	**Median follow up (months)**
Mohamed et al. (16) (American)	Non-RCT	SBRT	23	14/9	57.5 (44–70.2)	20/3	NR	50 (45–60)	17/0/5	41 (7.3–77.9)
		RFA	9	8/1	57.5 (44–70.2)	8/1	NR		9/0/0	41 (7.3–77.9)
Wahl et al. (10) (American)	Non-RCT	SBRT	63	49/14^*^	62 (35–85)	54/9^*^	2.2 (0–10)	50 (27–60)	43/18/2^*^	27 (0.5–86.5)^*^
		RFA	161	109/52^*^	60 (31–81)	117/44^*^	1.8 (0.6–7.0)		80/68/13^*^	50.9 (3.5–112.8)^*^
Sapisochin et al. (3) (Canada)	Non-RCT	SBRT	36	17/19^*^	60.4 (56.4–64.8)	30/5	4.5 (2.9–5.8)^*^	36 (30–40)	22/14/0^*^	28.1 (14.9–64.7)^*^
		RFA	244	156/88^*^	57.8 (53.5–62)	208/36	2.5 (1.9–3)^*^		158/68/8^*^	52.2 (21–90.7)^*^
Hara et al. (21) (Japan)	Non-RCT	SBRT	106	94/12	74 (48–93)	71/35	1.8 (1.0–3.0)	37.5 (35–40)	104/2/0	33.7 (0.5–75.0)
		RFA	106	93/13	75 (47–88)	76/30	1.7 (0.7–2.8)		105/1/0	29.9 (6.0–72.8)
Berger et al. (19) (American)	Non-RCT	SBRT	157	NR	68.61 (11.74)	113/44	4.8 (4.8)	NR	NR	NR
		RFA	627	NR	68.21 (10.00)	454/173	4.2 (4.6)		NR	NR
Duan et al. (22) (China)	Non-RCT	SBRT	37	NR	NR	NR	1–5	NR	NR	NR
		RFA	40	NR	NR	NR	1–5		NR	NR
Kim et al. (17) (Korea)	Non-RCT	SBRT	95	95/0	63.0 (35.0–85.0)	80/15	2.4 (0.7–5.5)	60 (52–60)	90/5/0	21.9 (11.8–31.2)
		RFA	95	95/0	67.0 (40.0–86.0)	83/12	2.1 (0.8–4.6)		90/5/0	21.6 (11.1–37.3)
Rajyaguru et al. (15) (American)	Non-RCT	SBRT	275	190/85	65 (55–75)	194/81	2.5 (2.5–3.5)	45 (45–55)	NR	25.3 (14.1–41.0)
		RFA	521	349/172	65 (55–75)	381/140	2.5 (2.5–3.5)		NR	25.3 (14.1–41.0)
Shiozawa et al. (16) (Japan)	Non-RCT	SBRT	35	35/0	75.1 (67–83)^*^	24/11	2.86 (1.2–5)^*^	50.6 (7.8)	28/7/0	12.6 (6.8–35.5)^*^
		RFA	38	38/0	68.7 (42–86)^*^	27/11	1.75 (0.7–2.9)^*^		31/7/0	18.7 (7.4–40.8)^*^
Parikh et al. (13) (American)	Non-RCT	SBRT	32	NR	77 (72–81.25)	20/12	NR	NR	NR	NR
		RFA	32	NR	79 (76–82)	22/10	NR		NR	NR
Total		SBRT	859							
		RFA	1,873							

*SBRT, stereotactic body radiotherapy; RFA, radiofrequency ablation; NR, not reported; RCT, randomized controlled trial*.

* *Statistically significant (P < 0.05)*.

### Local Progression Rates

**Figure 1:**
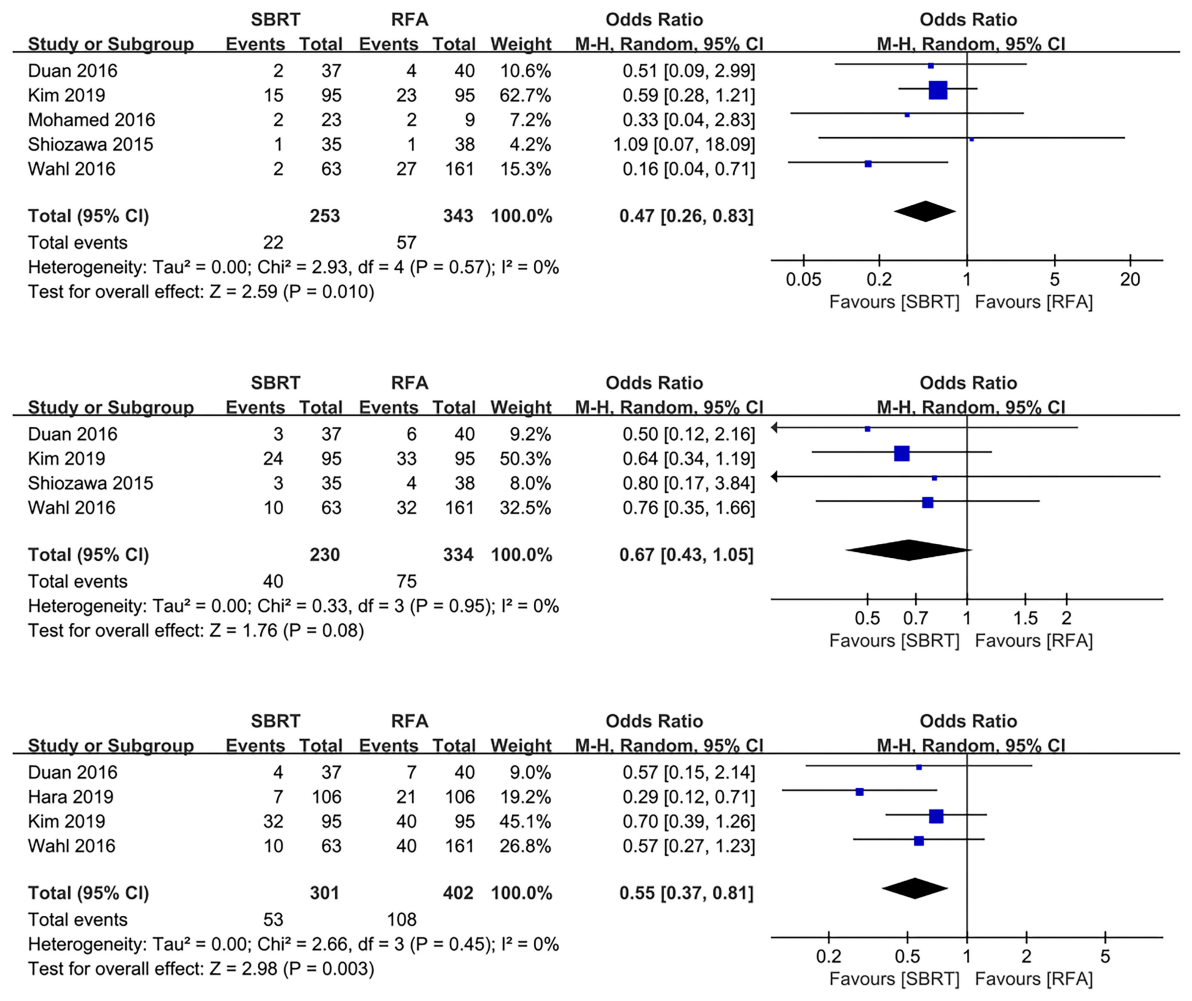
Forest plots demonstrating 1-, 2-, and 3-year LP in SBRT and RFA for HCC. LP, local progression; SBRT, stereotactic body radiotherapy; RFA, radiofrequency ablation; HCC, hepatocellular carcinoma.

Six out of the 10 studies illustrated 1-, 2-, and 3-year LP rates (10, 16–18, 21, 22). Our pooled results showed that the SBRT group had significantly better 1- and 3-year local control rates than the RFA group (OR 0.47, 95% CI 0.26–0.83, *P* = 0.010; and OR 0.55, 95% CI 0.37–0.81, *P* = 0.003, respectively). However, the 2-year LP rate showed marginal benefit of SBRT group than the RFA group (OR 0.67, 95% CI 0.43–1.05, *P* = 0.080). No heterogeneity was shown among the studies of 1-, 2-, and 3-year LP rates (χ^2^ = 2.93, I^2^ = 0%; χ^2^ = 0.33, *I*^2^ = 0%; and χ^2^ = 2.66, *I*^2^ = 0%, respectively) (Figure 1).

### Overall Survival

**Figure 2:**
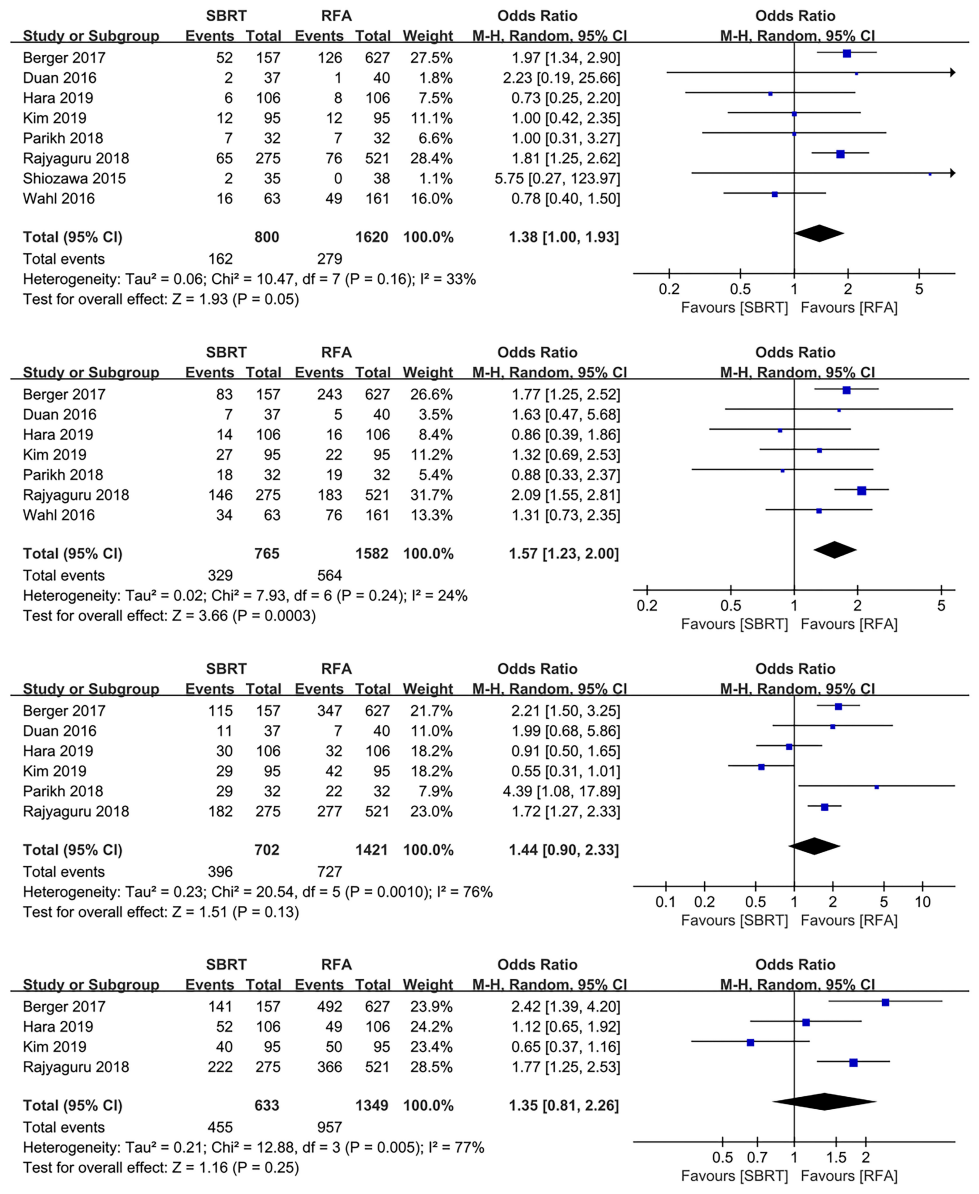
Forest plots demonstrating 1-, 2-, 3-, and 5-year LP in SBRT and RFA for HCC. LP, local progression; SBRT, stereotactic body radiotherapy; RFA, radiofrequency ablation; HCC, hepatocellular carcinoma.

Nine studies with 2,700 patients compared OS rates of SBRT group with RFA group (3, 10, 13, 15–17, 19, 21, 22), one study with 280 patients were excluded as both SBRT and RFA were applied as bridge therapies before transplantation, and the actual OS rates of SBRT or RFA might be affected by subsequent transplantation (3). The pool results showed that 2-year OS rates of RFA group were better than those of the SBRT group (OR 1.57, 95% CI 1.23–2.00, *P* < 0.0003), whereas there were no differences for 1-, 3-, and 5-year OS rates in both groups (OR 1.38, 95% CI 1.00–1.93, *P* = 0.050; OR 1.44, 95% CI 0.90–2.33, *P* = 0.130; and OR 1.35, 95% CI 0.81–2.26, *P* = 0.250, respectively; Figure 2).

Additionally, a secondary analysis was performed to control the potential report bias, and we enrolled the studies that reported outcomes of both LP and OS. As a result, the 1-, 2-, 3-, and 5-year OS rates indicated no significant difference between both groups (OR 0.96, 95% CI 0.59–1.57, *P* = 0.870; OR 1.35, 95% CI 0.89–2.03, *P* = 0.160; OR 0.97, 95% CI 0.28–3.36, *P* = 0.960; and OR 0.65, 95% CI 0.37–1.16, *P* = 0.150, respectively; Supplementary Figure 2).

### Prognosis for Treatment Allocation

**Figure 3:**
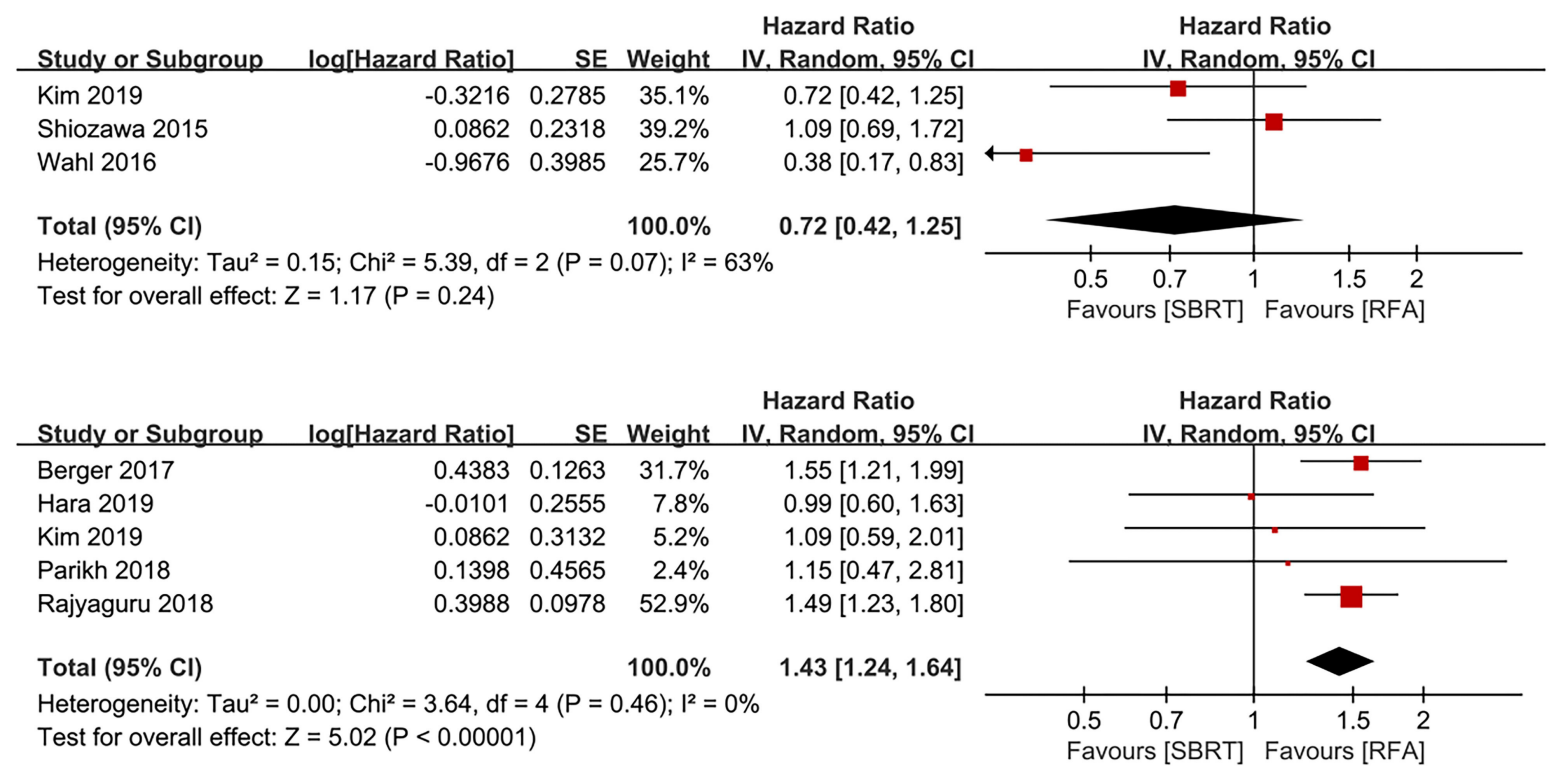
Forest plots demonstrating prognostic factor for LP and OS regarding to treatment allocation. LP, local progression; OS, overall survival.

Three and five studies evaluated the results of treatment allocation as a prognostic factor for LP (10, 16, 17) and OS (13, 15, 17, 19, 21), respectively. The treatment allocation was not a prognostic factor for LP (HR 0.72, 95% IC 0.42–1.25, *P* = 0.240). However, RFA group was more favorable than SBRT group for OS benefits (HR 1.43, 95% IC 1.24–1.64, *P* < 0.00001; Figure 3).

### Transplant and Post-transplant Pathological Necrosis Rate

**Figure 4:**
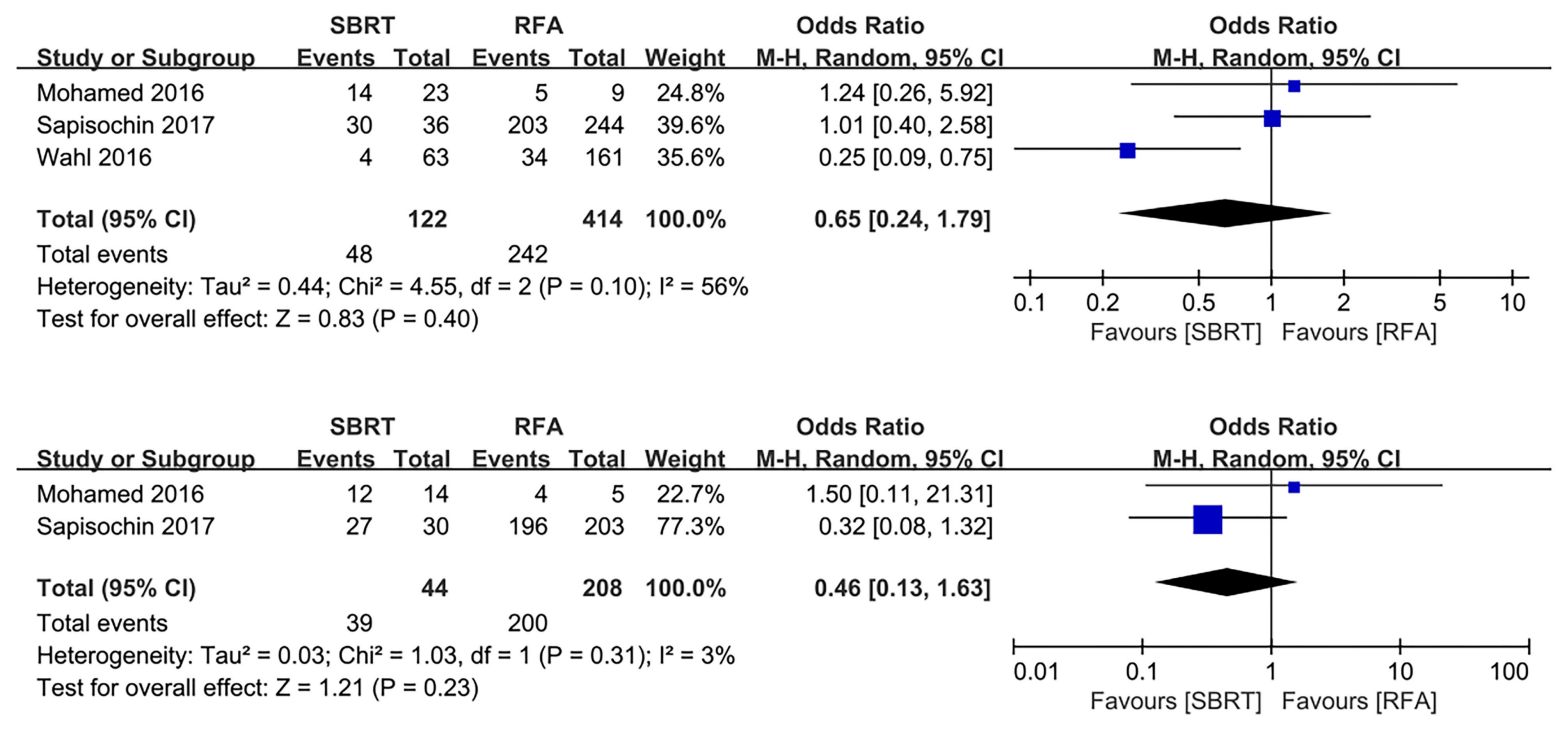
Forest plot demonstrating transplant rate and post-transplant pathologic necrosis in SBRT and RFA for HCC. SBRT, stereotactic body radiotherapy; RFA, radiofrequency ablation; HCC, hepatocellular carcinoma.

Three and two studies reported the transplant rate and post-transplant pathological necrosis rate, respectively (3, 10, 18). There were no significant differences in transplant rate and post-transplant pathological necrosis rate for both SBRT and RFA (OR 0.65, 95% CI 0.24–1.79, *P* = 0.040; and OR 0.46, 95% CI 0.13–1.63, *P* = 0.230, respectively; Figure 4).

### Publication Bias

The Begg funnel plot was used to evaluate the reliability of publication bias in our meta-analysis (25). The shape of 11 funnel plots was basically inverted and bilateral symmetry. Therefore, these results indicated that there was little publication bias among all comparisons in this meta-analysis (Supplementary Figure 3).

## Discussion

The main finding of this meta-analysis is that SBRT showed a better local control than RFA for patients with HCC, though the 2-year OS rates of SBRT were inferior because of the tumor burden and liver profiles. Recently, RFA, as a traditional curable treatment, was challenged by SBRT (15, 21, 22). With the development of imaging technique, such as four-dimensional CT (4DCT), SBRT is able to provide a more precise picture of HCC for treatment design (26). This improvement of SBRT effectively fixed the deficiencies of the high incomplete ablation rate of RFA under several specific conditions (7).

In 2006, Romero et al. (27) firstly applied the radiational technique of SBRT as a salvage treatment to control the primary and metastatic liver tumors. Although only 25 patients with 45 lesions that were unfit for other local control treatment were included in this study, the results preliminarily indicated that SBRT was feasible with acceptable toxicity and local control efficacy. They additionally pointed out that patients with Child B level were accompanied by high toxicity risks, and the optimal dose-fractionation schemes had to be found. Inspired by Romero et al., other investigators mainly focused on the application of SBRT on unresectable HCC and its effect in combination with other therapies (28–30). All these studies proved that SBRT was safe and provided satisfying local control for HCC, and this encouraged the temptation of expanding the indications of SBRT from a salvage or bridge treatment to curable intention.

RFA has long been applied as the first-line treatment for small HCC according to several clinical practice guidelines, including the European Society for Medical Oncology (ESMO) and American Association for the Study of Liver Disease (AASLD) (31, 32). However, RFA still suffers from high incidence of local incomplete ablation because of technical limitations [varies from 2 to 60% (5–9)], and it required additional or combinational therapies (33). As an advanced technique that shows reliable local control and safety on HCC, SBRT has been considered as a potential alternative therapy to RFA.

Many observational or retrospective studies in recent years have indicated that SBRT showed a non-inferior local control rate as compared with RFA (10, 16–18, 22). Shiozawa et al. (16) first compared the clinical outcomes between SBRT and RFA for HCC patients in a pilot study in 2015 and reported that SBRT was likely to become an important option for local treatment of early HCC. The subsequent studies indicated that SBRT appeared to be a reasonable alternative treatment of inoperable HCC in 2016 (10, 22). Subsequently, several large-volume validation studies based on online database had also proved comparable outcomes between SBRT and RFA regarding local control (13, 19). Moreover, in 2019, Kim et al. (17) retrospectively reviewed the institutional database for RFA and SBRT with curative intent, and they revealed that SBRT appears to be an effective alternative treatment for HCC when RFA is not feasible due to tumor location or size. However, another database validation from the American National Cancer Database revealed better OS of RFA than SBRT (15). A meta-analysis is needed to attain definitive proof to solve these debates. Thus, we performed this meta-analysis to help identify the advantages and disadvantages of SBRT and RFA in HCC.

In the present meta-analysis, with respect to local control, SBRT showed significantly lower 1- and 3-year LP rates, which indicated that SBRT achieved superior local control to RFA in treating HCC. In detail, there were five and four studies that enrolled the 1- and 3-year LP rate analyses, respectively. Among these studies, studies from Wahl et al. (10) and Duan et al. (22) were based on patients with inoperable HCC, studies of Shiozawa et al. (16) and Hara et al. (21) were based on patients with early-stage HCC, and studies from Kim et al. (17) and Mohamed et al. (18) did not specify patients characteristics. Most of the enrolled studies tended to draw the conclusions with respect to local control that supported SBRT. Moreover, Wahl et al. (10) and Hara et al. (21) reached significantly favorable results for SBRT in treating inoperable and early-stage HCC, respectively. However, only Kim et al. (17) clarified the information of HCC location; others did not refer to this critical factor, which might influence the local control of RFA in our previous study (34). Additionally, treatment allocation was not a significance prognostic factor on the basis of prognostic analysis. Therefore, further studies are needed to guarantee the appropriate individual treatment allocation.

Although SBRT enjoyed higher local control rates than RFA in the present study, the 2-year OS rates of SBRT were significantly lower than RFA. Notably, Berger et al. (19) and Rajyaguru et al. (15), who reported favorable OS rates of RFA with large sample volume, did not illustrate the LP rates correspondingly. There were two main reasons to the contradictory results between OS rates and LP rates. Firstly, there might be report bias when analyzing LP rates for both groups, which might result in inconsistent outcomes between LP rates and OS rates because of unreported LP. Therefore, we conducted the secondary analysis on OS rates that included the studies that reported both LP rates and OS rates, and we found that the 1-, 2-, 3-, and 5-year OS rates were comparable between SBRT and RFA (Supplementary Figure 2). Secondly, as the first-line treatment, RFA is more likely to be assigned to patients with better conditions. Patients who underwent SBRT were prone to suffer from larger tumor size and worse liver function, which indicated worse prognosis and decreased OS. Interestingly, the 3- and 5-year OS rates showed no significance in both groups, indicating that patients who did not die of tumor burden or liver functions in a short time might finally benefit from both treatments similarly. Meanwhile, RFA showed significant survival benefit for prognostic analysis on treatment allocation, which also could be explained by the reasons above. Therefore, we believed that the real effects of SBRT and RFA on long-term survival need to be further validated by high-level evidence, including RCTs.

As for bridge therapies to transplant, both SBRT and RFA provided a similar effect on patients waiting for transplantation. And the post-transplant pathological necrosis rate was comparable between both groups. These outcomes indicated that SBRT can be safely utilized as a bridge treatment to patients in the waiting list for transplantation with HCC, as an alternative to conventional bridging therapies. However, only three studies and 536 patients in total compared these parameters, and more solid studies were needed to be enrolled in the future.

This meta-analysis suffered from several limitations. First of all, only retrospective studies were available, resulting in relatively low quality of the evidence for the whole pooled results. Secondly, the results from this study should be interpreted carefully, because the sample sizes of four studies were relatively small, which is supposed to affect the reliability. And a further sensitivity analysis on the factors affecting outcomes could not be applied. Additionally, some studies were shorter follow-up in the SBRT group, which could result in obscuring late effects. Therefore, some well-designed, large, prospective, and multicenter studies are desperately needed to obtain more solid evidence.

## Conclusions

In sum, our meta-analysis shows that SBRT provided better local control than RFA, and it could be used as a potential alternative local control treatment for HCC.

## Author Contributions

Y-XP and D-DH designed the experiments and drafted the manuscript. Y-ZF, QL, J-CW, and MX were responsible for data collection and statistical analysis. S-LL, LX, and M-ZL helped revise the manuscript. M-SC and Y-JZ approved the final version. All authors contributed to the article and approved the submitted version.

## Conflict of Interest

The authors declare that the research was conducted in the absence of any commercial or financial relationships that could be construed as a potential conflict of interest.
