# Supplementary material for: Stereotactic Body Radiotherapy vs. Radiofrequency Ablation in the Treatment of Hepatocellular Carcinoma: A Meta-Analysis
Source: Front Oncol. 2020 Oct 29;10:1639. doi: 10.3389/fonc.2020.01639 (PMC7658324; doi:10.3389/fonc.2020.01639)
Supplement: Supplementary file 1 [file Table_1.docx]

| Supplementary Table S1 Risk of bias in retrospective studies using modified | | |  |  | |  | | | |  |  |  |
| --- | --- | --- | --- | --- | --- | --- | --- | --- | --- | --- | --- | --- |
|  | Selection | | | Comparability | | | | | Outcome | | |  |
| Study | Assignment for treatment | Representative treatment group | Representative reference group | Age | Sex | Tumor size | Liver Function | Tumor number | Assessment of outcome | | Adequate follow-up | Quality score |
| Mohamed et al., 2016 (American) | No | Yes | Yes | Yes | Yes | NR | Yes | Yes | Yes | | Yes | 6 |
| Wahl et al., 2016 (American) | No | Yes | Yes | Yes | No | Yes | No | No | Yes | | No | 5 |
| Sapisochin et al., 2017 (Canada) | No | Yes | Yes | Yes | Yes | No | No | No | Yes | | No | 4 |
| Hara et al., 2019 (Japan) | No | Yes | Yes | Yes | Yes | Yes | Yes | Yes | Yes | | Yes | 7 |
| Berger et al., 2017 (American) | No | Yes | Yes | Yes | Yes | Yes | NR | NR | Yes | | NR | 6 |
| Duan et al., 2016 (China) | No | Yes | Yes | NR | NR | Yes | NR | NR | Yes | | NR | 5 |
| Kim et al., 2019 (Korea) | No | Yes | Yes | Yes | Yes | Yes | Yes | Yes | Yes | | Yes | 7 |
| Rajyaguru et al., 2018 (American) | No | Yes | Yes | Yes | Yes | Yes | NR | Yes | Yes | | Yes | 6 |
| Shiozawa et al., 2015 (Japan) | No | Yes | Yes | No | Yes | No | Yes | Yes | Yes | | No | 5 |
| Parikh et al., 2018 (American) | No | Yes | Yes | Yes | Yes | NR | NR | NR | Yes | | NR | 6 |
| NR, not reported. | | | | | | | | | | | | |
