# Supplementary figures and images for: Stereotactic Body Radiotherapy vs. Radiofrequency Ablation in the Treatment of Hepatocellular Carcinoma: A Meta-Analysis
Source: Front Oncol. 2020 Oct 29;10:1639. doi: 10.3389/fonc.2020.01639 (PMC7658324; doi:10.3389/fonc.2020.01639)

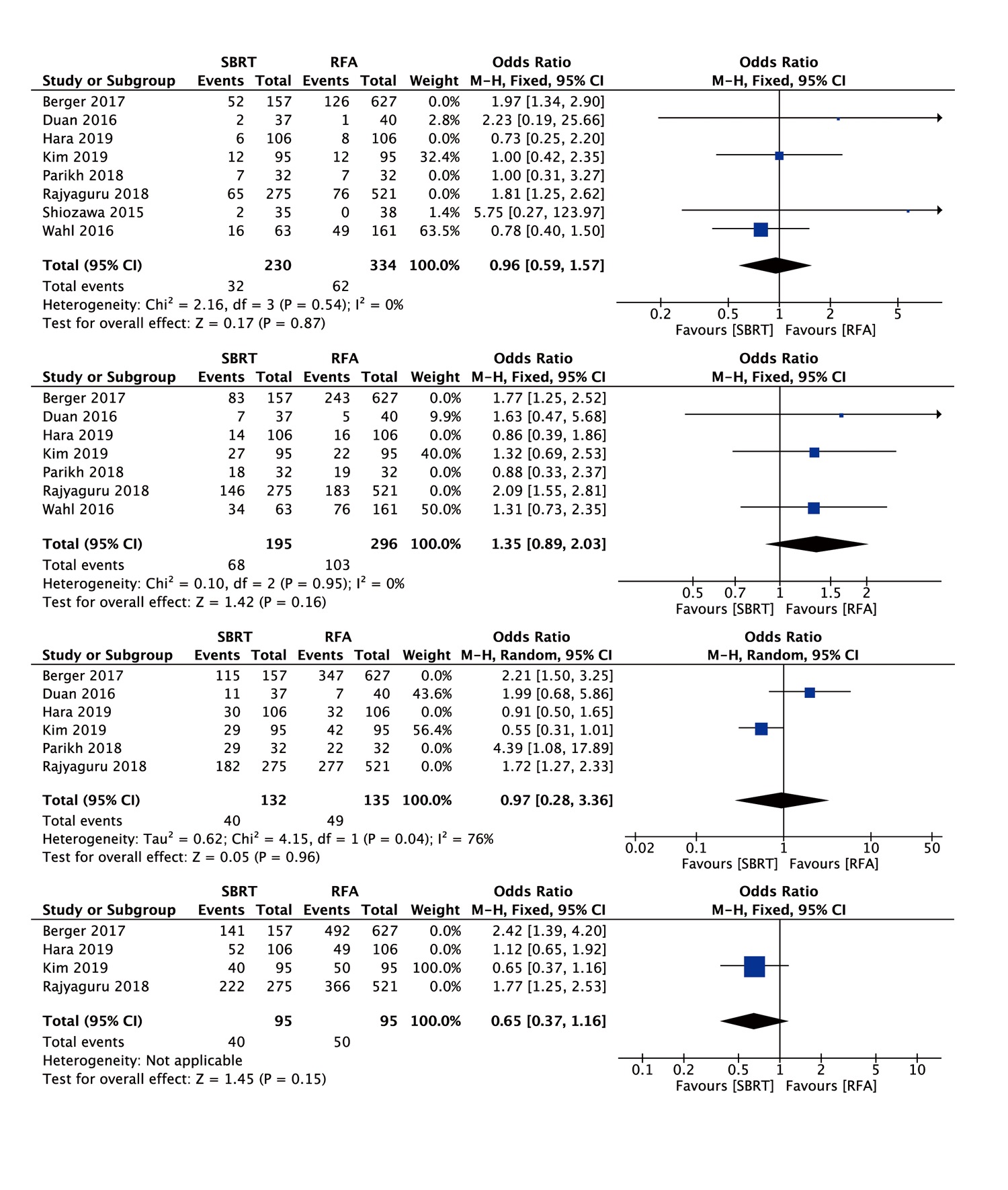

Supplement: Supplementary file 3 [file Image_2.JPEG]

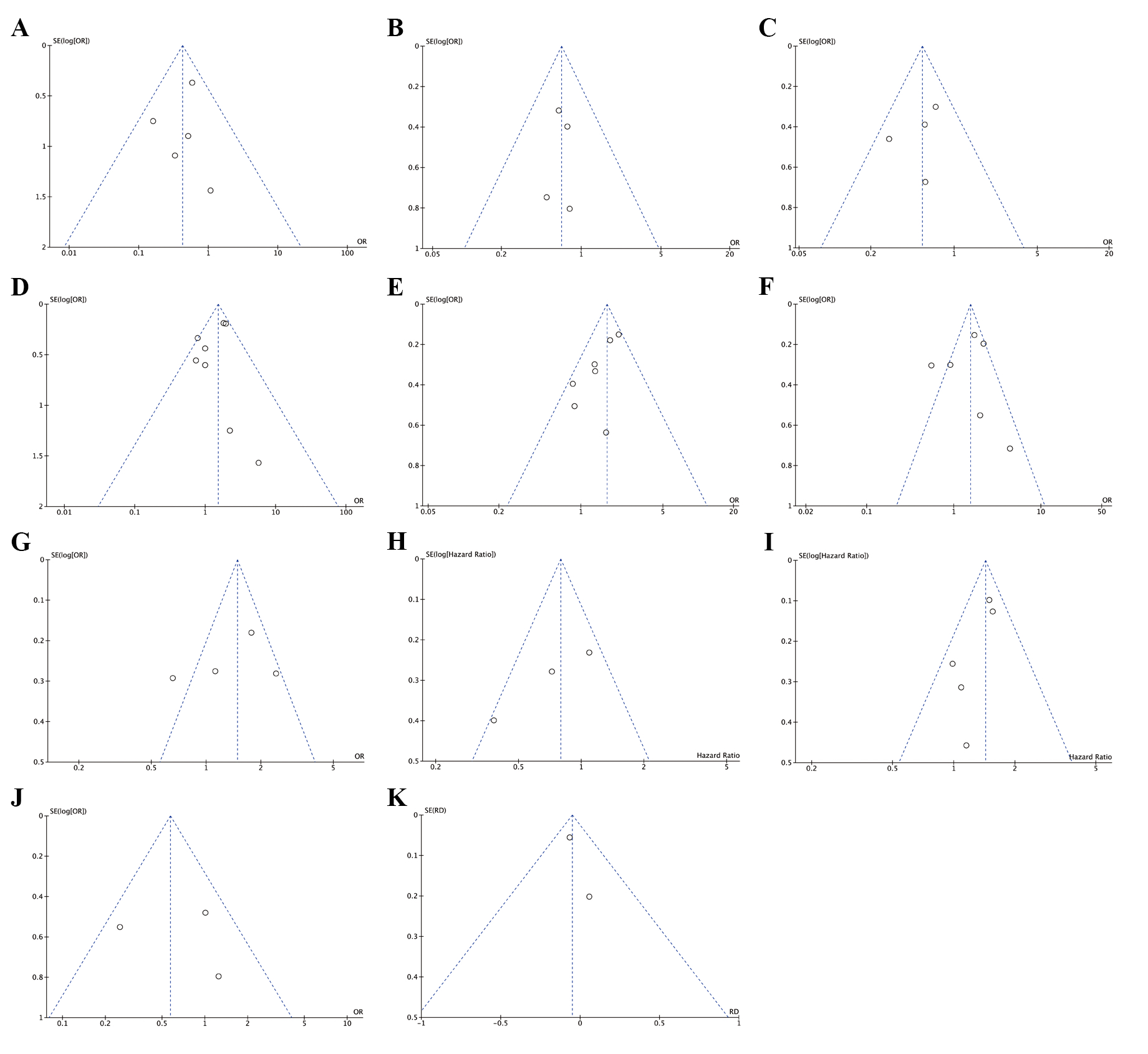

Supplement: Supplementary file 4 [file Image_3.JPEG]
